# The Colorectal cancer disease-specific transcriptome may facilitate the discovery of more biologically and clinically relevant information

**DOI:** 10.1186/1471-2407-10-687

**Published:** 2010-12-20

**Authors:** Wendy L Allen, Puthen V Jithesh, Gavin R Oliver, Irina Proutski, Daniel B Longley, Heinz-Josef Lenz, Vitali Proutski, Paul Harkin, Patrick G Johnston

**Affiliations:** 1 Centre for Cancer Research and Cell Biology, Queen's University Belfast, Belfast, BT9 7BL, Northern Ireland; 2 Almac Diagnostics Ltd, 19 Seagoe Industrial Estate, Craigavon, BT63 5QD, UK; 3 Division of Medical Oncology, University of Southern California/Norris Comprehensive Cancer Center, Keck School of Medicine, Los Angeles, California 90033, USA

## Abstract

**Background:**

To date, there are no clinically reliable predictive markers of response to the current treatment regimens for advanced colorectal cancer. The aim of the current study was to compare and assess the power of transcriptional profiling using a generic microarray and a disease-specific transcriptome-based microarray. We also examined the biological and clinical relevance of the disease-specific transcriptome.

**Methods:**

DNA microarray profiling was carried out on isogenic sensitive and 5-FU-resistant HCT116 colorectal cancer cell lines using the Affymetrix HG-U133 Plus2.0 array and the Almac Diagnostics Colorectal cancer disease specific Research tool. In addition, DNA microarray profiling was also carried out on pre-treatment metastatic colorectal cancer biopsies using the colorectal cancer disease specific Research tool. The two microarray platforms were compared based on detection of probesets and biological information.

**Results:**

The results demonstrated that the disease-specific transcriptome-based microarray was able to out-perform the generic genomic-based microarray on a number of levels including detection of transcripts and pathway analysis. In addition, the disease-specific microarray contains a high percentage of antisense transcripts and further analysis demonstrated that a number of these exist in sense:antisense pairs. Comparison between cell line models and metastatic CRC patient biopsies further demonstrated that a number of the identified sense:antisense pairs were also detected in CRC patient biopsies, suggesting potential clinical relevance.

**Conclusions:**

Analysis from our *in vitro *and clinical experiments has demonstrated that many transcripts exist in sense:antisense pairs including *IGF2BP2*, which may have a direct regulatory function in the context of colorectal cancer. While the functional relevance of the antisense transcripts has been established by many studies, their functional role is currently unclear; however, the numbers that have been detected by the disease-specific microarray would suggest that they may be important regulatory transcripts. This study has demonstrated the power of a disease-specific transcriptome-based approach and highlighted the potential novel biologically and clinically relevant information that is gained when using such a methodology.

## Background

Response rates for advanced colorectal cancer (CRC) remain disappointingly low at 40-50% for 5-FU-based combination therapies [1,2]. The poor response rates are due to drug resistance, which is either inherent or acquired in nature. A number of predictive markers of response to these therapies have been proposed, however, the results are controversial [3-16] and to date, outside of KRAS testing, no predictive markers have made the transition to routine clinical use. Due to the lack of clinical implementation of molecular markers there is a need to identify robust predictive markers of response to ultimately increase response rates to treatment in these patients.

Many studies have identified predictive markers or cassettes of predictive markers using gene expression measurements [3,17-21]. Within the current study we have utilized the leading generic microarray and compared it to a disease-specific transcriptome-based microarray. It is of interest to assess the content of the unique information present in the disease-specific microarray in relation to drug treatment and in the identification of potential predictive markers in this disease setting. Recently, the ENCODE pilot project published its findings on the detailed characterization of 1% of the human genome [22]. The study observed a much higher level of transcription than was originally thought to occur including a high level of non-protein encoding transcripts. Indeed several studies have suggested that up to 20% of all protein-encoding genes could have an associated natural antisense transcript (NAT) [23]. The aim of the present study was to assess the benefit of a disease-specific transcriptome-based profiling approach compared to a generic genomic-based microarray. In addition, we examined the composition of the disease-specific transcripts and found a high level of NAT expression both *in vitro *and clinically in this disease setting. These have a functional role in response to drug treatment in colorectal cancer and warrant further investigation.

## Methods

### Microarray Profiling and Experiment Design

We have previously carried out microarray profiling experiments using HCT116 colorectal cancer cells on the Affymetrix HGU133 Plus2.0 array (Plus2.0 array) [24] and the Almac Diagnostics Colorectal cancer DSA (Colorectal DSA). HCT116 parental cells and 5-FU-resistant daughter cells [25] were either untreated (0 h control) or treated with 5 μM 5-FU (IC_50(72 h) _for the parental cell line) for 24 hours (Additional File 1A). The comparison between parental control and parental treated with 5-FU is referred as the 'sensitive' experiment; while the comparison between the resistant control and the resistant treated with 5-FU is referred as the 'resistant' experiment. Microarray profiling was carried out on 28 pre-treatment (Irinotecan/5-FU) metastatic biopsies using the Colorectal DSA [26]. All patients provided written fully informed consent as per IRB guidelines in the University of Southern California and approval was granted from this body. These patients underwent biopsy of colorectal liver metastases prior to commencing irinotecan/5-FU chemotherapy on the IFL schedule. Detailed experimental protocols and raw expression data are available at http://www.ebi.ac.uk/arrayexpress/ (Accession numbers E-MEXP-1691 (*in vitro) and *E-MEXP-1692 (Clinical) for Colorectal DSA analysis and Accession number E-MEXP-390 for Affymetrix Plus2.0 analysis).

### Quantitative reverse transcription-PCR analysis

Total RNA was isolated using RNA STAT-60 (Tel-Test, Inc.) according to the manufacturer's instructions. Reverse transcription was carried out using 2 μg of RNA using a Moloney murine leukemia virus-based reverse transcriptase kit (Invitrogen) according to the manufacturer's instructions. Quantitative reverse transcription-PCR (RT-PCR) amplification was carried out in a final volume of 10 μL containing 5 μL of 2×SYBR green master mix (Qiagen), 4 μL of primers (2 μM), and 1 μl of cDNA using an Opticon DNA Engine Thermal Cycler (Bio-Rad Laboratories, Inc., Waltham, MA) using methods previously described [26]. All amplifications were primed by pairs of chemically synthesized 18- to 22-mer oligonucleotides designed using freely available primer design software (Primer3) http://frodo.wi.mit.edu/primer3/ (Additional file 2).

### Derivation of unique microarray content lists

HG-U133 Plus2.0 full sequences and probes were downloaded from the Affymetrix website http://www.affymetrix.com/ in FASTA format. Probe and full sequences used in the design of the Colorectal DSA were obtained from Almac Diagnostics in FASTA format.

Probe sequences from the Colorectal DSA probesets were aligned against the Plus2.0 array full length sequences using BLAST [27]. Where 6 or more probes from a probeset (usually 11 probes) aligned to the same sequence with 100% identity over their entire length, the DSA probeset and the Affymetrix sequence were considered 'common'. Full length sequences representing the DSA probesets not considered common at this stage were extracted and the Plus2.0 array probesets were BLASTed against them. Where 6 or more probes from a probeset (usually 11 probes) aligned to the same sequence with 100% identity over their entire length, the DSA sequence and the Affymetrix probeset were again considered 'common'. Those sequences/probesets not considered common at this stage also formed the 'unique' groupings.

### Data Analysis

Data analysis was conducted using either Genespring GX v 7.3.1 (Agilent Technologies, UK) or the R statistical package [28] and Bioconductor [29]. Background correction, scaling and summarization of the raw data to generate expression values were done with the MAS5 algorithm. The experiment was setup to measure the ability of each microarray platform to detect probesets, detect differentially expressed probesets and also to detect biologically relevant (cancer-related) probesets.

#### Detection Filtering

The detection of probesets was measured based on the MAS5 present, marginal and absent flag calls. For all the replicates, probesets passing the flag call filter as present or marginal were counted using data from the whole microarrays in both Plus2.0 array and Colorectal DSA. The number of probesets consistently detected across the 3 replicates in each condition, i.e., untreated parental, 5-FU treated parental, untreated 5-FU resistant and 5-FU treated 5-FU resistant, was calculated by selecting the probesets passing the flag filter in all the 3 replicates in each case.

#### Differential Expression Filtering

For both the Colorectal DSA and the Plus2.0 complete microarray data, following detection filtering, probesets were further filtered based on fold change in expression and a statistical filter in the case of both HCT116 parental and 5-FU resistant cell line data. Differential expression was measured between untreated and 5-FU treated samples in both the sensitive and resistant experiments. All probesets passing the fold change filter of 1.3 fold and also with a t-test p-value less than 0.05 were counted for differentially expressed transcripts.

### Pathway Analysis

All pathway analysis was carried out using Genespring v7.3.1 (Agilent Technologies, UK) using both KEGG and GenMAPP pathways. Pathway analysis was carried out using the complete content of each microarray platform for those probesets that were detected (present/marginal) and differentially expressed (1.3-fold + t test) in the sensitive and resistant experiments and pathways were selected that contained greater than 15 genes (sensitive experiment) and 10 genes (resistant experiment) per pathway. Statistical analysis for each pathway was carried out using hypergeometric statistics. The number of genes per pathway cut-off was selected based on the total number of genes contained within a given experiment.

#### Analysis of sense and antisense probesets

The total number of sense and antisense transcripts in the unique content (23,089 probesets) of the Colorectal DSA was assessed. In addition to the Colorectal DSA-specific probesets, the numbers of sense and antisense probesets within this group, which were detected in the *in vitro *(sensitive and resistant) and clinical experiments were also assessed independently. Detection was determined by the present and marginal flag calls as described earlier. Finally, probesets that passed both the detection filter and differential expression filter were classified into sense and antisense orientations and counted

In all the cases above, it was further investigated to find whether sense:antisense (SAS) pairs exist

### Genomic Alignment of SAS pairs

Full sequences corresponding to the Colorectal DSA probesets were aligned to the human genome (Ensembl release 51.36 m; NCBI build 36) using BLAT via the Ensembl [30] website http://www.ensembl.org/index.html. The highest scoring alignments were viewed using the 'region in detail' view of the Ensembl genome browser. Tracks were customized to include known Ensembl genes and GENSCAN [31] predicted genes using the 'configure this page' option.

### Clinical Analysis

All probesets from the colorectal DSA analysis of metastatic CRC patient samples were initially filtered using detection flag calls, with present or marginal calls in > 50% of all samples. Subsequently, probesets were filtered using differential expression with a change of 1.5-fold in at least one condition (CR, PR, SD and PD). Sense and antisense probes were isolated from each list and only the probes with associated annotation were taken forward for SAS pair analysis.

## Results

### Comparison of the Affymetrix HGU133 Plus2.0 microarray with the Almac Diagnostics Colorectal Cancer DSA

To assess the benefit of the Almac Diagnostics Colorectal Cancer Disease-Specific Array (further referred as "Colorectal DSA") we compared it directly to the Affymetrix Human Genome U133 Plus2.0 microarray (further referred as "Plus2.0 array"). In this study, the HCT116 colorectal cancer cell line was used along with the 5-FU-resistant daughter cell line [25]. The HCT116 parental (5-FU sensitive) cell line was either untreated (control) or treated with 5 μM 5-FU for 24 h, and will be referred further as the "sensitive experiment". The HCT116 5-FU-resistant cell line was either untreated (control) or treated with 5 μM 5-FU for 24 h, and will be referred as the "resistant experiment". RNA was harvested and arrayed in triplicate (biological replicates) on either the Colorectal DSA or the Plus2.0 array (Additional File 1A). When comparing the Colorectal DSA to the Plus2.0 array, the Colorectal DSA contained 61,528 probesets in total, with 23,089 probesets specific (unique) to the Colorectal DSA. The Plus2.0 array contained 54,675 probesets, of which 24,941 probesets were specific (unique) to the Plus2.0 array (Table 1).

**Table 1:** Content of Plus2.0 array and Colorectal DSA

Probesets	HGU133 Plus2.0	Colorectal DSA
Total	54,675	61,528

Common	29,734	38,439

Unique	24,941	23,089

In addition to the total content of the microarrays, the number of probesets in common, as well as, the number of Colorectal DSA-specific probesets and Plus2.0-specific probesets are also displayed in the table.

To compare the two microarray platforms, we compared the complete content of each array based on detection (Affymetrix MAS5 present (P) or marginal (M) flag calls) and detection + differential expression (1.3-fold change and t-test p-value <0.05).

### Validation of *In Vitro *Microarray Analyses

In order to validate the microarray results, we measured the expression of a representative number of genes from the *in vitro *Colorectal DSA experiment by quantitative RT-PCR, we have previously validated the Plus2.0 array experiment [24]. For the Colorectal DSA 13 genes (Additional file 2) were selected for validation; all validations were carried out in three independent experiments (Additional file 3). The genes were selected based on fold-induction, with both highly and more moderately induced genes chosen and both up-regulated and down-regulated genes analyzed.

For the selected genes, the average fold-changes by both microarray and quantitative RT-PCR were log transformed and the correlation between the expression values were examined using Pearson's product correlation moment (r). For the 13 genes acutely altered in the HCT116 parental cells following 5-FU treatment over 24 h, the Pearson's correlation (r) was 0.75, with r^2 ^= 0.57 (p = 0.0032). In terms of the basal alterations between parental and 5-FU-resistant cells, Pearson's correlation (r) of the 13 genes was 0.78, with r^2 ^= 0.61 (p = 0.0017) (Additional file 3). Taken together, these results demonstrate that there is a strong overall concordance between the real-time PCR validation and the microarray experiment. Therefore these results highlight the robustness of the original microarray experiment.

### Analysis of the total content of the microarray platforms

**Figure 1:**
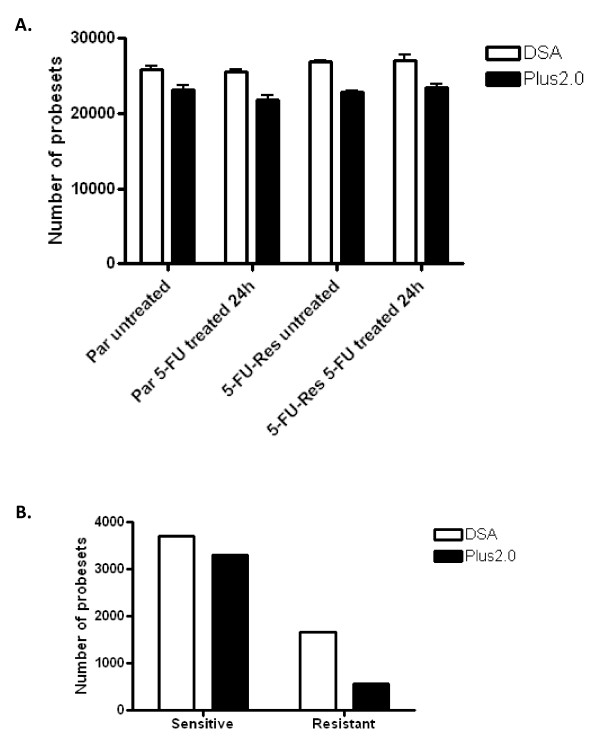
**Complete probeset analysis**. Results from the analysis of the complete content of the Plus2.0 array and the Colorectal DSA. **A. **Graph showing the number of probesets consistently detected (P or M) across all three replicates of parental untreated, parental 5-FU treated, 5-FU-resistant untreated and 5-FU-resistant 5-FU treated. **B. **Graph displaying the number of probesets detected, differentially expressed (at 1.3-fold) and passing t-test filter (p < 0.05) in the parental and 5-FU-resistant cells following 5-FU treatment.

The total content of each microarray platform was assessed based on detection and detection plus differential expression (Figure 1A-B). When comparing the Colorectal DSA to the Plus2.0 array based on detection only, the Colorectal DSA consistently detected (in all three replicates) a higher number of present or marginal probesets compared to the Plus2.0 array (Figure 1A). There was also a marked reduction in the variance across the replicates for the colorectal DSA compared to the Plus2.0 array suggesting a greater degree of reproducibility for the Colorectal DSA.

The complete content of each microarray was also compared based on detection and differential expression. The colorectal DSA detected a higher number of probesets compared to the Plus2.0 array when detection and differential expression were taken into account. The colorectal DSA detected 3713 differentially expressed probesets in the sensitive and 1660 differentially expressed probesets in the resistant experiments while the Plus2.0 array detected only 3296 differentially expressed probesets in the sensitive and 564 differentially expressed probesets in the resistant experiments (Figure 1B). Taken together, these results suggest that the Colorectal DSA consistently detects a higher number of differentially expressed probesets and displays a lower variance between sample replicates.

### Pathway analysis of the microarray platforms

To further assess the biological relevance of each microarray platform, we carried out pathway analysis on the probesets that passed the detection filter, 1.3-fold change and also t test filtering (p-value < 0.05) in the sensitive experiment. Using all of the probesets of each microarray as a starting point, the Plus2.0 array had 3,296 probesets that passed all three filters while the Colorectal DSA had 3,713 probesets that passed all three filters in the sensitive experiment. Following pathway analysis, filtered data from the Plus2.0 array generated 10 statistically significant differentially regulated pathways (Table 2). Starting with the filtered data from the Colorectal DSA 16 statistically significant differentially regulated pathways were identified (Table 3). Overall there were 7 pathways in common between the two microarray platforms: cell cycle, folate biosynthesis, glycerophospholipid metabolism, oxidative phosphorylation, purine metabolism, pyrimidine metabolism and starch and sucrose metabolism (Tables 2 and 3). When examining the common pathways between the two platforms in terms of the number of probesets detected and differentially expressed, the Colorectal DSA detected significantly more probesets for 6 of the 7 pathways (Cell cycle, Glycerophospholipid metabolism, Oxidative phosphorylation, Purine metabolism, Pyrimidine metabolism and Starch and sucrose metabolism). The Plus2.0 array only detected more probesets for one of the common pathways (Folate biosynthesis). The pathways that were identified by the Plus2.0 array alone were Aminoacyl-tRNA biosynthesis, Ubiquitin-mediated proteolysis and Wnt signaling, while the pathways identified by the Colorectal DSA only were Biosynthesis of steroids, DNA polymerase, Fatty acid metabolism, Fructose and mannose metabolism, Glycolysis and Gluconeogenesis, Insulin signaling pathway, Proteasome, Tryptophan metabolism and Valine, leucine and isoleucine degradation.

**Table 2:** Pathway analysis from Plus2.0 array

Pathways	Number of genes	p value
Aminoacyl-tRNA biosynthesis	18	1.50×10^-07^

Cell cycle	47	6.67×10^-05^

Folate biosynthesis	19	2.44×10^-06^

Glycerophospholipid metabolism	19	0.0158

Oxidative phosphorylation	25	0.0176

Purine metabolism	50	2.86×10^-07^

Pyrimidine metabolism	33	6.26×10^-07^

Starch and sucrose metabolism	21	0.000845

Ubiquitin mediated proteolysis	21	0.00116

Wnt signaling pathway	42	0.0453

Based on the complete content of the Plus2.0 array for 3296 genes passing flags, 1.3-fold change and t-test in the sensitive experiment. Displayed are the numbers of genes from a given pathway that are detected, differentially expressed and also passing a t-test filter. Pathways selected that are statistically significant based on hypergeometric statistics.

**Table 3:** Pathway analysis from Colorectal DSA

Pathways	Number of genes	p value
Biosynthesis of steroids	17	1.26×10^-07^

Cell cycle	50	1.24×10^-05^

DNA polymerase	16	0.000153

Fatty acid metabolism	17	0.0119

Folate biosynthesis	18	0.000637

Fructose and mannose metabolism	18	0.00818

Glycerophospholipid metabolism	21	0.0271

Glycolysis Gluconeogenesis	17	0.00872

Insulin signaling pathway	44	0.0427

Oxidative phosphorylation	46	2.98×10^-09^

Proteasome	15	1.50×10^-08^

Purine metabolism	59	8.06×10^-07^

Pyrimidine metabolism	43	3.15×10^-08^

Starch and sucrose metabolism	26	0.000391

Tryptophan metabolism	27	0.00542

Valine, leucine and isoleucine degradation	19	0.00915

Based on the complete content of the Colorectal DSA for 3713 genes passing flags, 1.3-fold change and t-test in the sensitive experiment. Displayed are the numbers of genes from a given pathway that are detected, differentially expressed and also passing a t-test filter. Pathways selected that are statistically significant based on hypergeometric statistics.

We also examined which pathways were differentially regulated in the resistant experiment between the Plus2.0 array and the Colorectal DSA. In the resistant experiment, following filtering (Flags, 1.3-fold and t-test), 1660 genes were identified as altered following 5-FU treatment using the Colorectal DSA, while only 564 genes were identified as altered following 5-FU treatment using the Plus2.0 array. Pathway analysis revealed that 19 pathways were altered following 5-FU treatment using the Colorectal DSA, while only 3 pathways were altered following 5-FU treatment using the Plus2.0 array (Additional File 4). The 3 pathways (Focal adhesion, MAPK signaling and regulation of the actin cytoskeleton) that were identified using the Plus2.0 array were also identified using the Colorectal DSA, therefore, the Plus2.0 array was not identifying any unique information. In addition, there was no overlap in the identified pathways between the sensitive and the resistant experiments using the Plus2.0 array. Using the Colorectal DSA, 16 unique pathways were identified that were not identified by the Plus2.0 array and 4 pathways (Cell cycle, Insulin signaling, Purine metabolism and Pyrimidine metabolism) were identified in both the sensitive and the resistant experiments (Additional File 5). These pathways may play an important role not only in drug response, but also in drug resistance. Overall, it appears that compared to the Plus2.0 array, the Colorectal DSA is providing more biologically relevant information, both in the sensitive and resistant experiments.

### Composition of the specific Colorectal DSA content

**Figure 2:**
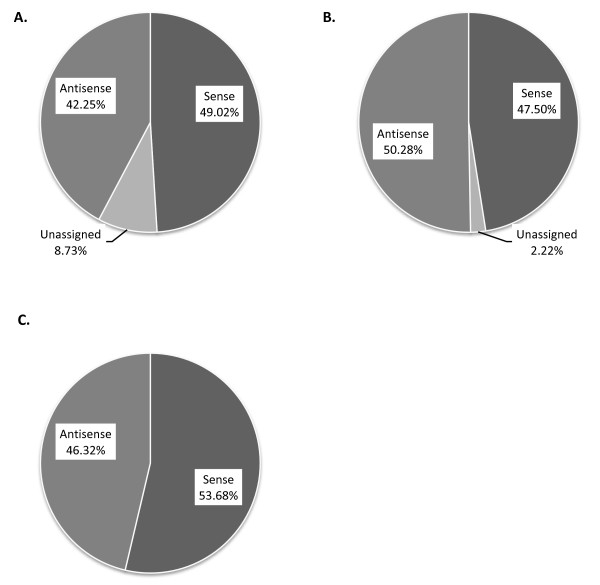
**Colorectal DSA-specific probeset analysis**. Pie charts displaying the Colorectal DSA-specific (unique) content (probesets) breakdown for the 5-FU sensitive experiment. **A. **Total Colorectal DSA-specific content breakdown. **B. **Based on detected probesets. **C. **Based on detection + differential expression.

The specific content (23,089 probesets) of the Colorectal DSA was investigated to assess which groups of probesets predominated following detection +/- differential expression filtering. When the specific content was assessed without any filtering applied, 11,320 (49.02%) probesets were in the sense orientation, 9,754 (42.25%) probesets were in the antisense orientation and 2,015 (8.73%) had no orientation assigned (Figure 2A). Following detection filtering in the sensitive experiment, 771 (47.5%) probesets were in the sense orientation, 816 (50.28%) were in the antisense orientation and only 36 (2.22%) had no orientation assigned (Figure 2B). Finally, 102 (53.68%) probesets were in the sense orientation and 88 (46.32%) probesets were in the antisense orientation following detection plus differential expression filtering in the sensitive experiment (Figure 2C). Similar results were obtained for the resistant experiment (Additional File 6).

Due to the predominance of probesets in antisense orientation following filtering in the sensitive and resistant experiments, and the fact that little is known about the functionality of these 'antisense' transcripts we chose to investigate them further. Starting with the sense probesets and antisense probesets, we isolated only those with corresponding Unigene ID and removed the probesets with redundant Unigene IDs. Out of the 6,073 sense probesets and 5,324 antisense probesets 2,456 were in sense:antisense (SAS) pairs. In the sensitive experiment, following detection filtering, 45 probesets were matched SAS pairs, with 661 antisense probesets and 638 unique sense probesets. Following detection and differential expression filtering in the sensitive experiment, 9 probesets were matched SAS pairs, with 234 antisense probesets and 267 sense probesets (Table 4). Similar results were found for the resistant experiment (Table 4). Overall, for the sensitive experiments, up to 7% of the sense and antisense probesets existed in SAS pairs, while up to 9% of the sense and antisense probesets existed in SAS pairs in the resistant experiment.

**Table 4:** Sense and antisense in vitro analysis

DSA-specific probesets	Sense	Antisense	SAS pairs
All probesets	6073	5324	2456

Sensitive experiment Detection	638	661	45

Resistant experiment Detection	724	760	68

Sensitive experiment Detection + DE	267	234	9

Resistant experiment Detection + DE	215	223	8

Table displaying the number of genes (DSA-specific) in the sense and antisense orientation for the 5-FU sensitive and resistant *in vitro *experiment. Also displayed is the total number of probesets that are represented in both the sense and antisense orientation (SAS pairs). All analyses include genes identified from detection +/- differential expression filtering. Acronyms used: DSA: Disease-specific array, SAS: sense:antisense pairs, Detection: probesets passing Affymetrix flag calls, DE: differential expression.

### Sense:Antisense (SAS) probe pair analysis

#### In vitro analysis

Of the 1299 Colorectal DSA-specific probesets detected in this experiment, 661 were in the antisense orientation and 638 were in the sense orientation, and 45 were common to both sense and antisense probesets and termed SAS probe pairs (Additional File 6). Gene ontology analysis revealed that the SAS probesets were involved in a plethora of biological processes, of those the most statistically robust terms were oxidative phosphorylation, JAK-STAT signaling, phosphorylation, metabolism, cell death and splicing (data not shown).

**Figure 3:**
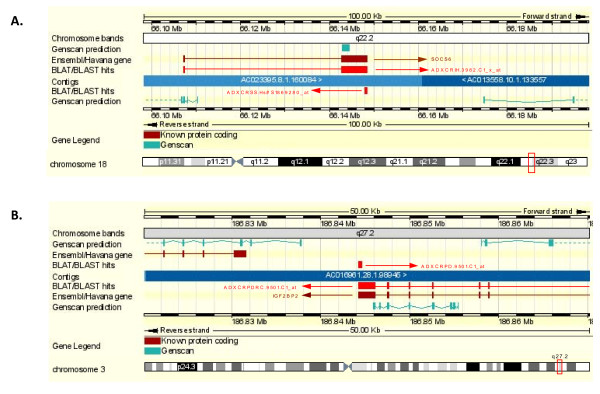
**Genomic alignments**. Genomic alignments for two of the SAS pairs represented on the Colorectal DSA and passing detection filtering in the 5-FU sensitive experiment. Sequences aligning to the sense strand of the genome appear in the upper half of the images while sequences aligning to the antisense strand of the genome appear in the lower half. A. Alignment of the DSA sequences corresponding to the SOCS6 gene. B. Alignment of the DSA sequences corresponding to the IGF2BP2 gene.

We then chose two SAS probesets randomly from the list of 45 as exemplars for sequence alignment, which were SOCS6 and IGF2BP2. We aligned the full length sequences of the sense and antisense probesets to the human genome via the ENSEMBL website. The full length sequence for the SOCS6 sense probeset aligns exactly with the SOCS6 gene, while the full length sequence of the antisense probeset for SOCS6 is located on the reverse strand of SOCS6 and shows clear sequence overlap with the full length SOCS6 sequence (Figure 3A). The second SAS probeset that was chosen for further analysis was IGF2BP2. Again, we aligned our full length sense and antisense sequences to the human genome and found that the sense IGF2BP2 full length sequence aligned exactly with the IGF2BP2 gene, which was located on the reverse strand. In addition, our antisense full length sequence was located on the forward strand and it displayed good sequence overlap with the full length sense sequence (Figure 3B). The occurrence and more importantly, the altered expression of these SAS pairs would suggest that the antisense sequences may have some functional role in this disease setting, which may be in gene regulation.

#### Clinical analysis

Due to the high level of Colorectal DSA probesets observed in the antisense orientation and given the results obtained from the *in vitro *analysis (Table 4) we wanted to assess if there were similar levels of antisense expression in clinical colorectal tumour samples, and in addition, if any of these probesets were expressed in SAS pairs. Microarray profiling was carried out on 28 pre-treatment (Irinotecan/5-FU) metastatic biopsies using the Colorectal DSA. In this analysis, the annotated probesets from the specific content of the Colorectal DSA were isolated for further examination. It was observed that, following detection filtering, 265 probesets occurred in the sense orientation, 168 probesets occurred in the antisense orientation and 8 probesets existed in SAS pairs (Table 5). In addition, following detection and differential expression filtering, 87 probesets occurred in the sense orientation, 67 probesets occurred in the antisense orientation and 3 occurred in SAS pairs (Table 5). Interestingly, the analysis from the pre-treatment metastatic biopsies demonstrated that there is a high incidence of antisense expression in clinical samples and furthermore, that up to ~5% of these occur in SAS pairs suggesting a potential functional role and in addition, clinical relevance of these SAS pairs. Of note, one of the SAS pairs identified from the pre-treatment metastatic biopsies was also identified from the *in vitro *analysis (both 5-FU-sensitive and -resistant experiments), the probeset coded for the *IGF2BP2 *gene.

**Table 5:** Sense and antisense clinical analysis

DSA-specific probesets	Sense	Antisense	SAS pairs
All probesets	6073	5324	2456

Detection	265	168	8

Detection + DE	87	67	3

Table displaying the number of genes (DSA-specific) in the sense and antisense orientation for the pre-treatment metastatic CRC biopsies. Also displayed is the total number of genes that are represented in both the sense and antisense orientation (SAS pairs). All analyses include genes identified by detection +/-differential expression filtering. Acronyms used: DSA: Disease-specific array, SAS: sense:antisense pairs, Detection: probesets passing Affymetrix flag calls, DE: differential expression.

When comparing the probesets in the sense orientation, antisense orientation and those that exist in SAS pairs, it was observed that 244 sense probesets are common between the sensitive *in vitro *and clinical experiments, while 247 sense probesets are common between the resistant *in vitro *and clinical experiments and 565 sense probesets were found to be common between the sensitive and resistant *in vitro *experiments. Further analysis demonstrated that 147 antisense probesets were shared between the sensitive *in vitro *and clinical experiments, 150 antisense probesets were common between the resistant *in vitro *and clinical experiments, while 582 antisense probesets were common between the sensitive and resistant *in vitro *experiments. Finally, in terms of those probesets that were detected as SAS pairs, 7 were common between the sensitive *in vitro *and clinical experiments, 5 SAS pairs were shared between the resistant *in vitro *and clinical experiments and 34 SAS pairs were common between the sensitive and resistant *in vitro *experiments (Additional File 7).

## Discussion

The aim of this study was to compare transcriptional profiling data generated from colorectal cancer cell lines following treatment with 5-FU using either a leading generic genomic-based microarray (Plus2.0 array) or a disease-specific transcriptomic-based microarray (Colorectal DSA). The Colorectal DSA was developed based on the colorectal transcriptome, which was generated from large-scale in-house sequencing, public data mining and experimental investigation [32]. The DSA array is a transcriptome based array as opposed to the Plus 2.0 which a genomic based array. Given the greater complexity of the transcriptome in comparison to the genome, it would be expected that an array of this type would detect a greater number of transcripts. When comparing the Colorectal DSA to the Plus2.0 array, the Colorectal DSA contains 37.5% unique information (23,089 probesets), which is not contained on the Plus2.0 array and the aim of the current study was to assess how important this unique information is. One of the benefits of the Colorectal DSA is that it is also based on the Affymetrix GeneChip technology meaning that cross-platform comparisons are possible.

The same experimental design was used for each microarray study, consisting of parental or 5-FU-resistant HCT116 cells either untreated or treated with 5-FU for 24 h. The resultant expression profile generated from the parental cells following treatment with 5-FU was termed as the sensitive experiment, while the expression profile generated from the resistant cells following 5-FU was termed as the resistant experiment. To assess the performance of each microarray platform we compared the complete content (all probesets) of the arrays based on detection (Flags, present or marginal) and detection plus differential expression.

Following analysis of the complete content of the microarrays, the Colorectal DSA outperformed the Plus2.0 array in terms of probesets detected and detected plus differentially expressed and also displayed a lower variance between sample replicates. In addition, the Colorectal DSA identified more pathways in both the sensitive and the resistant experiments when compared to the Plus2.0 array and also identified common pathways important for drug response and also drug resistance, cell cycle, insulin signaling, purine metabolism and pyrimidine metabolism. Indeed, it is not surprising that cell cycle, purine and pyrimidine metabolism pathways were altered following 5-FU treatment in sensitive and 5-FU-resistant cells given the mechanism of action of the drug. Interestingly, insulin signaling was also altered following 5-FU treatment in both sensitive and resistant settings. Previous studies have demonstrated that insulin signaling has an important role in colorectal cancer progression [33,34]. Dallas *et al *demonstrated that colorectal cancer cells that are resistant to 5-FU and oxaliplatin, by repeated exposure to drug, are more responsive to IGF-1R inhibition than the parental cells [35], suggesting that insulin signaling is deregulated during the process of acquiring drug resistance. There are a number of reasons that can account of the observed differences in pathway identification between the two platforms, firstly, in terms of the 'complete' probeset analysis, the Colorectal DSA detected more probesets and also more differentially expressed probesets than the Plus2.0 array. More importantly, in terms of those probesets that are unique to each array platform our analysis suggested that the Plus2.0 array detected more probesets than the Colorectal DSA. In terms of pathway analysis we are interested in specific genes, so when we assessed the percentage of probesets that coded for a single gene name, we found that the Colorectal DSA identified many more individual genes than the Plus2.0 array, which identified multiple probesets that coded for the same gene name. Overall, this suggests that the Colorectal DSA was identifying more differentially expressed 'unique' genes than the Plus2.0 array and this accounts for the observed differences in pathway identification between the two array platforms.

We also wanted to examine the microarray specific content of the Colorectal DSA, which was not present on the Plus2.0 array. We found that approximately 50% of the Colorectal DSA specific probesets are in the antisense orientation, which is much higher than expected. Upon further examination of the microarray-specific probesets, we demonstrated that some are expressed in either the sense or antisense orientations only, while a portion (up to 8.9%) are detected in sense:antisense (SAS) pairs. Recently, the publication of the ENCODE pilot project, which aimed to provide a detailed characterization of 1% of the human genome, demonstrated that there is a much higher level of transcription than originally thought and this includes the generation of a high number of non-protein encoding transcripts [36]. In addition, the literature suggests that approximately 20% of human protein-encoding genes have an associated natural antisense transcript (NAT), however, recent studies suggest that this figure could be much higher [23,37-40]. NATs can be divided into either cis-acting or trans acting in nature [41]. Cis-acting NATs are transcribed from the opposing DNA strand at the same genomic locus, while trans-acting NATs are transcribed from separate loci. The cis-NATs can also be further categorized according to their relative orientation and degree of overlap, either 5' to 5' (head to head), 3' to 3' (tail to tail) or fully overlapping [37,41]. NATs have been proposed to regulate the expression of their target genes at several levels, but as yet no experimental data has been provided to assign a definite function to NATs. However, some studies using RT-PCR, northern blotting or microarray profiling have validated the expression of antisense transcripts [23,38,39,42]. Interestingly, some SAS pairs are flanked by the same transcription factor binding sites, suggesting that the SAS pairs may be co-regulated [41]. Analysis has demonstrated that SAS pairs can display concordant expression patterns, or discordant expression patterns [37]. In addition, studies have demonstrated that targeting an antisense transcript using a siRNA approach can alter the levels of the sense transcript, by either up-regulating sense transcription or down-regulating sense transcription [40,43], so the results are not always as expected. However, the same studies have demonstrated that alterations of the sense transcript does not affect the antisense expression levels [40,43].

As previously described, the functional role of these antisense transcripts is currently unknown, but they have been implicated in transcriptional and translational interference, RNA masking, dsRNA-dependent mechanisms, alternative splicing, stability, cellular transport and chromatin remodeling [37,40,41,44]. However, the functional relevance of antisense transcripts is something that is now commonly accepted [45-47]. Studies have demonstrated that long antisense transcripts function as epigenetic regulators of transcription in human cells [46]. In addition, studies that have validated the functional relevance of antisense transcripts suggest that they are not a uniform group of regulatory RNAs, but rather that they carry out a wide variety of biological roles [47]. The utility of a transcriptome-based approach has been demonstrated in the detection of these non-coding antisense transcripts, as this information could be important when examining pathway regulation. Further examination of these NATs may answer a number of important questions such as why when an upstream regulator of a pathway is highly up regulated at the mRNA level do we not see downstream mediators up regulated, or why do the changes observed at the RNA level not always correlate with protein expression? Obviously, a great deal of experimental work would need to take place to assess whether NATS do play a role in gene regulation, but if as we suspect at least some do, we need to not only examine the sense transcripts, but also the antisense transcripts at the same time to get a true view of what is happening in the cell, for example, following drug treatment.

We further examined the 45 SAS pairs that were detected as either present or marginal in the 5-FU sensitive experiment; we decided not to include a fold change filter at this stage as it is not necessarily to have both the sense and the antisense transcript altered to a certain level to see a functional effect. For example, the antisense may be up regulated which leads to the suppression of the sense, resulting in no change in the sense probeset. Overall, when we examined the intensities/expression of the probesets contained within the SAS pairs it was found that ~50% displayed similar intensities, therefore displaying no differential intensities between sense and antisense probesets. However, ~50% displayed discordant or differential intensities, therefore this group of SAS pairs may be the most functionally relevant, however, this will require more experimental testing. Gene ontology analysis demonstrated that these SAS pairs were involved in diverse biological processes, with the most statistically robust involved in oxidative phosphorylation, JAK-STAT signaling, phosphorylation, metabolism, cell death and splicing. We further chose two SAS pairs to examine at the sequence level, they were *SOCS6 *and *IGF2BP2*. Sequence alignment demonstrated that the full length *SOCS6 *transcript aligned exactly with the *SOCS6 *gene on the forward strand of chromosome 18. In addition, the full length antisense transcript aligned to the reverse strand of chromosome 18 and demonstrated good tail to tail sequence overlap with the full length sense sequence and the *SOCS6 *gene. In terms of *IGF2BP2*, the full length sense sequence aligned completely with the *IGF2BP2 *gene on the reverse strand of chromosome 3. The full length antisense sequence aligned to the forward strand of chromosome 3 and again demonstrated good tail to tail overlap with the full length sense sequence and the *IGF2BP2 *gene. The sequence alignment results demonstrate that the SAS pairs show good overlap in sequence and appear to be cis-NATS that are transcribed from the opposing DNA strand in the same genomic locus. Numerous novel SAS pairs have previously been identified on DSA microarrays and their existence validated with alternative technologies including strand-specific RT-PCR. Functional relevance has also been suggested through analysis of SAS pair expression patterns [48]. Full characterization of the IGF2BP2 and SOCS6 antisense transcripts will require further work which forms the basis of future studies however; inspection of the sequences with the Ensembl Human Genome Browser supports their existence. Extensive EST evidence exists and appears to suggest a regular exonic structure. Numerous currently unclassified regulatory elements also occur in the region surrounding the sequences. Since both the EST sequencing used in DSA design and the experimental labelling process are polyA-based, it would suggest that the transcripts are polyadenylated, but since the ESTs represent only a fragment of the full transcript, analysis of precise polyA signal location and constitution (i.e. canonical or non canonical) is difficult.

To investigate the clinical relevance of SAS pairs we utilized microarray data generated from pre-treatment (irinotecan/5-FU) metastatic colorectal biopsies with full response data. Following detection filtering we demonstrated that 8 SAS pairs existed (4.8% of total antisense and 3% of total sense probesets). In addition, we demonstrated that 3 SAS pairs existed following detection plus differential expression filtering (4.5% total antisense and 3.4% total sense probesets). Upon examination of the probesets in the sense orientation, antisense orientation and those existing in SAS pairs between *in vitro *experiments and clinical experiments, the results demonstrate that there is a high percentage of sense, antisense and SAS pairs that exist between *in vitro *and clinical samples. The clinical experiments generated fewer sense, antisense and SAS pairs than the *in vitro *experiments, however, a high percentage of those detected in the clinical experiment were also detected in the *in vitro *experiments. Taken together, these results suggest that *in vitro *experiments do highlight potentially clinically relevant information; however, these types of analysis would require further independent validation. These *in vitro *and clinical analyses demonstrate in this disease setting that potentially up to 8.9% of all probesets could exist in SAS pairs; currently there is little investigation to the functional role that these SAS pairs may play. Interestingly, one SAS pair, *IGF2BP2*, was found to be common between the *in vitro *and the clinical analysis. IGF2BP2 has been demonstrated to regulate translation of IGF2 by binding to its 5'UTR [49]. In addition, IGF2 is known to be overexpressed in cancer [50,51] and specifically, insulin signaling has been demonstrated to play a role in colorectal cancer [35,52-55]. Given the results from the pathway analysis also identifying the significance of insulin signaling, further experimental investigation into the identified SAS pairs, in particular *IGFBP2*, should discover if some or all have functional relevance in this disease setting and whether they are disease-specific or have more widespread effects. The focus of future studies examining the SAS pairs identified from this study will also include questions such as what is their exact function within the cell, are they all functioning in the same way in this disease setting or is it dependent on the specific SAS pair.

One of the limitations of this analysis is that we compared the power of the two microarray platforms using data generated from a single 5-FU-sensitive and -resistant cell line model. While the main focus of the study was to directly compare the data generated from the two microarray platforms based on detected transcripts and pathways and for this a single model cell line would be appropriate, however, a secondary aim was to assess the biological relevance of the colorectal transcriptome and compare this to a generic genomic approach. In this respect the use of a number of CRC cell line models would have given greater insight into the power of such an approach as the problem of tissue homogeneity would have been addressed to some degree. It is widely accepted that cell lines models are not very representative of the primary tumour and to somewhat address these issues we identified the unique biological information, SAS pairs, that was generated using the colorectal transcriptome-based approach and assessed if these occurred in metastatic (liver) CRC patient biopsies. The cell line models identified 45 SAS pairs and when we examined the data generated from the clinical biopsies we found that not as many SAS pairs existed, 8 in total were detected. When we compared the SAS pairs from the cell lines and patient biopsies we found that 7 were in common, therefore ~87% of the clinical SAS pairs were also contained within the cell line SAS pairs list. This would suggest that many of the cell line SAS pairs are lost in the clinical samples probably due to the homogeneity of the cell line model and that those are occurring in the clinical samples may be the most biologically relevant, however, further analysis of these SAS pairs would be required.

## Conclusions

In conclusion, we have carried out transcriptional profiling using the Plus2.0 array and the Colorectal DSA and compared their overall performance. We observed that the transcriptome-based Colorectal DSA has outperformed the genome-based Plus2.0 array as demonstrated by the detection and differential expression of the entire microarray content. This study has demonstrated that the strength of a disease-specific transcriptome-based approach is in the amount of biologically relevant information gained, as noted from the pathway analysis. When analyzing the results from the Colorectal DSA a number of pathways, cell cycle, insulin signaling, purine metabolism and pyrimidine metabolism, were highlighted as important regulators of drug response and drug resistance, which were not identified using the Plus2.0 array. In addition, the novel biologically relevant information gained from the Colorectal DSA contained a number of antisense probesets that exist in SAS pairs, including *IGF2BP2*, again highlighting the potential importance of insulin signaling, also highlighted by pathway analysis. It is currently unclear at this point what the functionality of the identified NATs is, but the literature suggests that they may be involved in diverse gene regulatory mechanisms. However, it is clear from the numbers of antisense probesets detected and differentially expressed by the Colorectal DSA that these may be very important regulatory transcripts. Finally, if this disease-specific transcriptome-based approach was not utilized in this setting, important biologically relevant information, including the regulation of SAS pairs could potentially be overlooked.

## Abbreviations

The following abbreviation were used throughtout the text, CRC: Colorectal Cancer; 5-FU: 5-Fluorouracil; DSA: Disease specific array; NAT: Natural antisense transcript; SAS: Sense:antisense.

## Competing interests

Professor Patrick Johnston and Prof Paul Harkin are the Founders and Directors of Almac Diagnostics, Craigavon, UK. Gavin Oliver and Vitali Proutski are employees of Almac Diagnostics, Craigavon, UK.

## Authors' contributions

WLA was involved in the conception and design of the study, the acquisition, analysis and interpretation of the data and drafted the manuscript, PVJ was involved in the conception and design of the study, the analysis and interpretation of the data and helped draft the manuscript, GRO carried out the sequence alignments and revised the manuscript critically for important intellectual content; IP carried out the microarray QPCR validations and revised the manuscript critically for important intellectual content; DBL revised the manuscript critically for important intellectual content, HJL revised the manuscript critically for important intellectual content, VP was involved in the conception and design of the study and revised the manuscript critically for important intellectual content, DPH revised the manuscript critically for important intellectual content, and PGJ conceived the study, and participated in its design and coordination and helped to draft the manuscript. All authors read and approved the final manuscript.

## Pre-publication history

The pre-publication history for this paper can be accessed here:

http://www.biomedcentral.com/1471-2407/10/687/prepub

## Supplementary Material

Additional file 1 **A. Microarray experimental design**. HCT116 parental and 5-FU-resistant daughter cells were either untreated or treated with an IC_50 _dose (of parental) of 5-FU for 24 h. All microarrays were run in triplicate (biological replicates) on the Plus2.0 array and the Colorectal DSA. The sensitive experiment is defined as those transcriptional changes following 5-FU treatment in the parental setting, while the resistant experiment is defined as those transcriptional changes following 5-FU in the 5-FU-resistant setting. Click here for file

Additional file 2 **Quantitative RT-PCR primer sequences for Colorectal DSA microarray validation**. Click here for file

Additional file 3 **Graphs showing microarray results and quantitative RT-PCR validations for 13 genes selected from the Colorectal DSA**. All data was log transformed and the Pearson's correlation calculated for (A) the parental HCT116 cells following treatment with 5-FU for 24 h and (B) the basal comparison between the HCT116 parental and the 5-FU-resistant sub line. All experiments were carried out in triplicate (biological replicates). Click here for file

Additional file 4 **Pathway analysis based on the resistant experiment for the complete content of the Plus2.0 array and the Colorectal DSA**. In the Plus2.0 array experiment 564 genes pass flags, 1.3-fold change and t test filtering in the resistant experiment. In the Colorectal DSA experiment 1660 genes pass flags, 1.3-fold change and t test filtering in the resistant experiment. Pathways selected that contain more than 10 genes per pathway. Click here for file

Additional file 5 **Table displaying the 45 SAS pairs that were identified from the sensitive experiment following detection filtering**. Displayed is gene name, Unigene ID, Entrez gene ID and gene description. Click here for file

Additional file 6 **Pie charts displaying the Colorectal DSA-specific (unique) content (probesets) breakdown for the 5-FU-resistant experiment**. **A. **Based on detected probesets. **B. **Based on detection + differential expression. Click here for file

Additional file 7 **Table displaying the number of detected sense, antisense and SAS pairs, from the DSA unique content, that are common between the sensitive and resistant *in vitro *experiments, the sensitive *in vitro *and clinical experiments and the resistant *in vitro *and clinical experiments**. Click here for file
