# Supplementary material for: The Colorectal cancer disease-specific transcriptome may facilitate the discovery of more biologically and clinically relevant information
Source: BMC Cancer. 2010 Dec 20;10:687. doi: 10.1186/1471-2407-10-687 (PMC3018462; doi:10.1186/1471-2407-10-687)
Supplement: Additional file 1 — A. Microarray experimental design. HCT116 parental and 5-FU-resistant daughter cells were either untreated or treated with an IC50 dose (of parental) of 5-FU for 24 h. All microarrays were run in triplicate (biological replicates) on the Plus2.0 array and the Colorectal DSA. The sensitive experiment is defined as those transcriptional changes following 5-FU treatment in the parental setting, while the resistant experiment is defined as those transcriptional changes following 5-FU in the 5-FU-resistant setting. [file 1471-2407-10-687-S1.PPTX]

## Slide 1
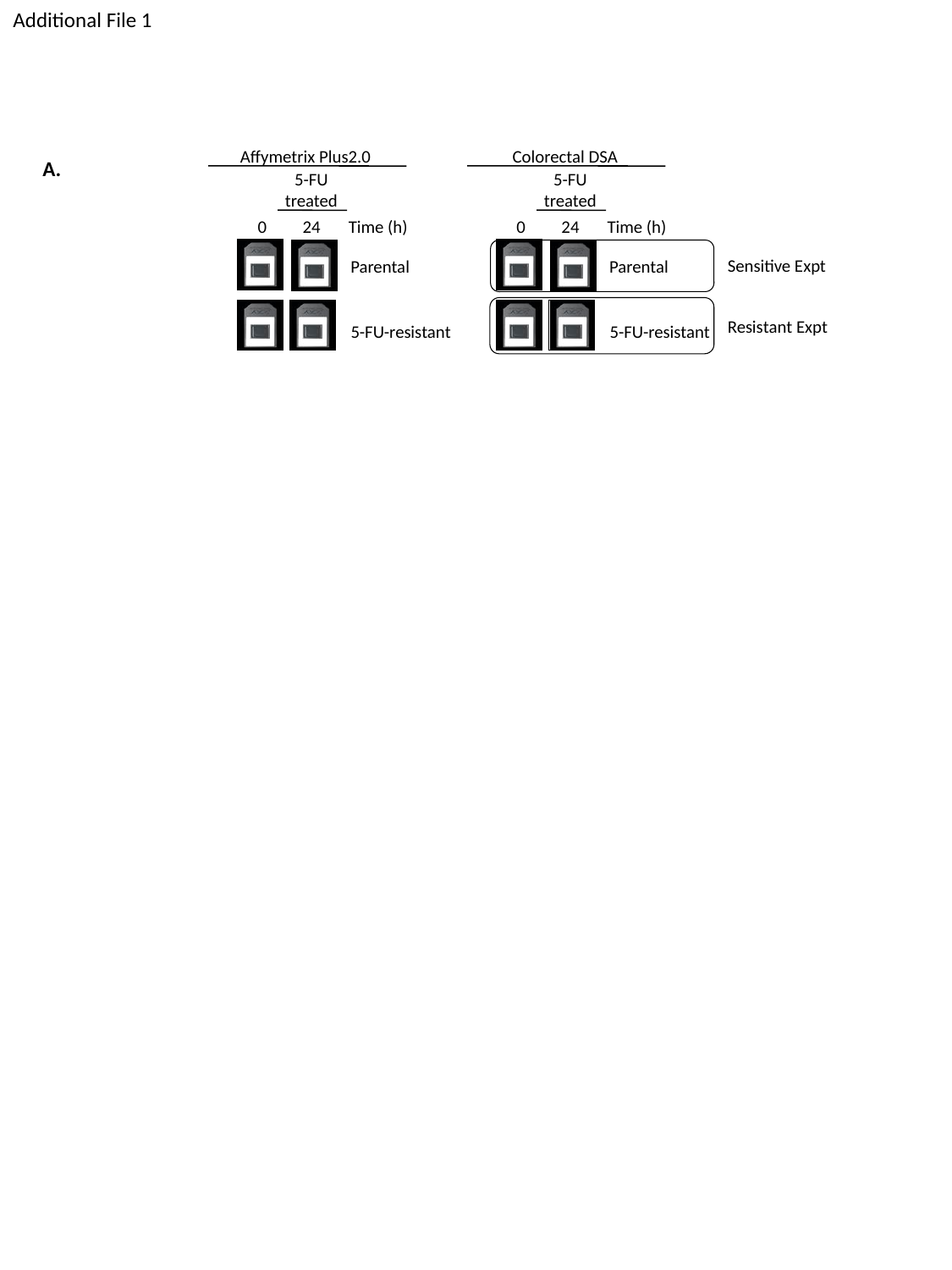

Additional File 1
Affymetrix Plus2.0
Colorectal DSA
A.
5-FU
treated
5-FU
treated
0 24 Time (h)
0 24 Time (h)
Sensitive Expt
Parental
Parental
Resistant Expt
5-FU-resistant
5-FU-resistant
