# Supplementary figures and images for: The Colorectal cancer disease-specific transcriptome may facilitate the discovery of more biologically and clinically relevant information
Source: BMC Cancer. 2010 Dec 20;10:687. doi: 10.1186/1471-2407-10-687 (PMC3018462; doi:10.1186/1471-2407-10-687)

## Slide 1
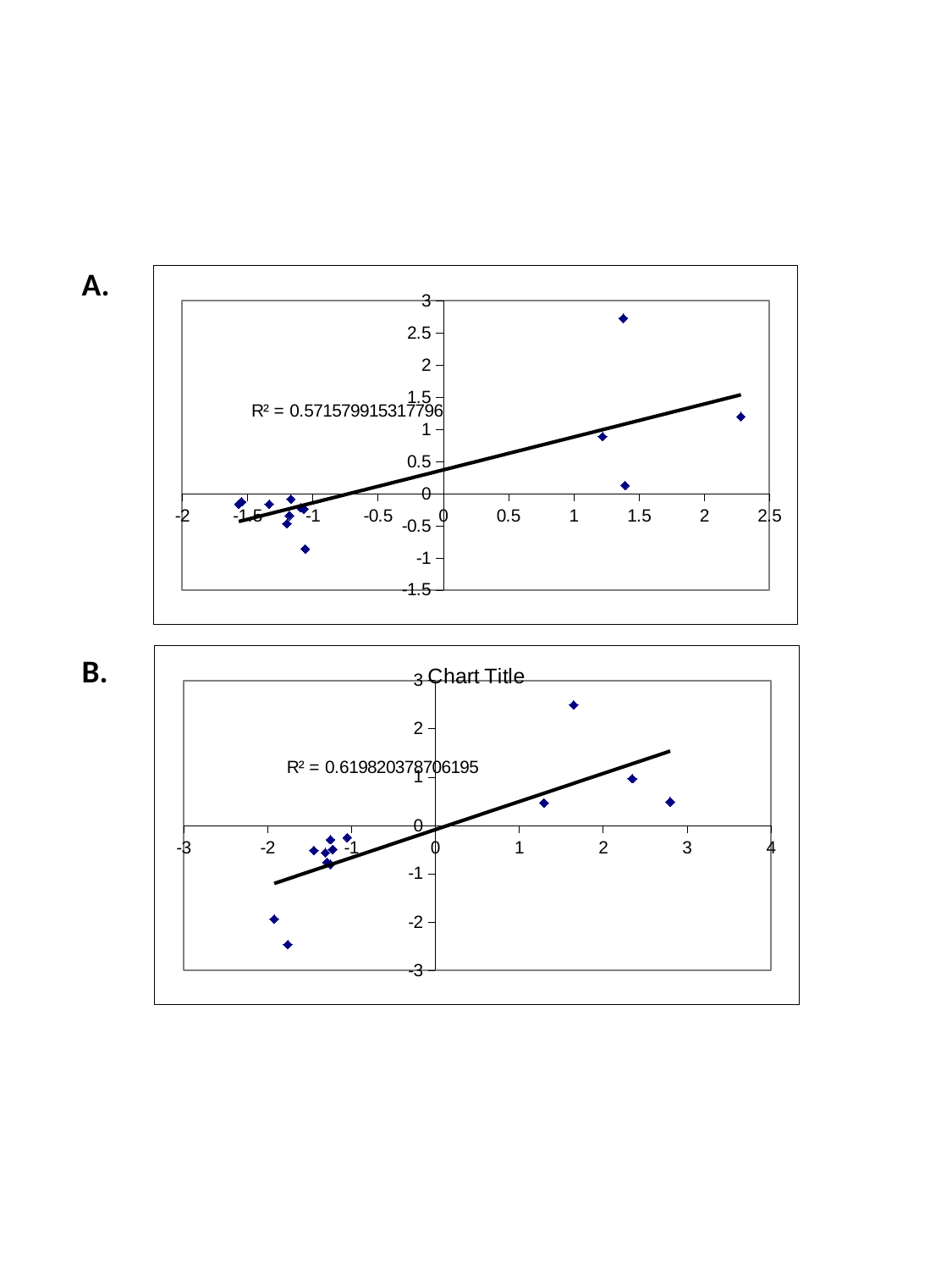

A.
### Chart
| Category | |
|---|---|
### Chart:
| Category | |
|---|---|B.

Supplement: Additional file 3 — Graphs showing microarray results and quantitative RT-PCR validations for 13 genes selected from the Colorectal DSA. All data was log transformed and the Pearson's correlation calculated for (A) the parental HCT116 cells following treatment with 5-FU for 24 h and (B) the basal comparison between the HCT116 parental and the 5-FU-resistant sub line. All experiments were carried out in triplicate (biological replicates). [file 1471-2407-10-687-S3.PPTX]
