# Supplementary material for: The Colorectal cancer disease-specific transcriptome may facilitate the discovery of more biologically and clinically relevant information
Source: BMC Cancer. 2010 Dec 20;10:687. doi: 10.1186/1471-2407-10-687 (PMC3018462; doi:10.1186/1471-2407-10-687)
Supplement: Additional file 4 — Pathway analysis based on the resistant experiment for the complete content of the Plus2.0 array and the Colorectal DSA. In the Plus2.0 array experiment 564 genes pass flags, 1.3-fold change and t test filtering in the resistant experiment. In the Colorectal DSA experiment 1660 genes pass flags, 1.3-fold change and t test filtering in the resistant experiment. Pathways selected that contain more than 10 genes per pathway. [file 1471-2407-10-687-S4.PPTX]

## Slide 1
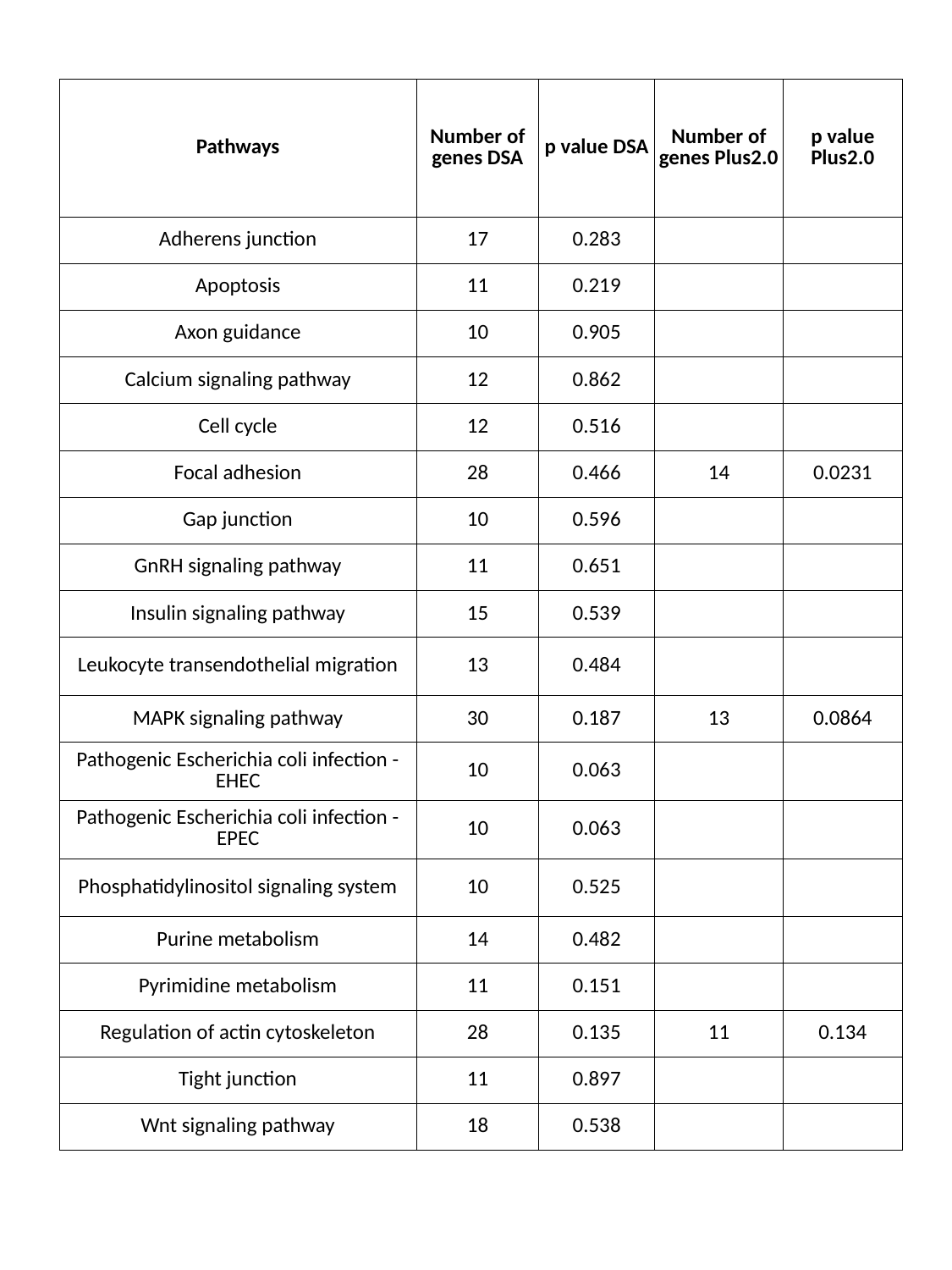

| Pathways | Number of genes DSA | p value DSA | Number of genes Plus2.0 | p value Plus2.0 |
| --- | --- | --- | --- | --- |
| Adherens junction | 17 | 0.283 | | |
| Apoptosis | 11 | 0.219 | | |
| Axon guidance | 10 | 0.905 | | |
| Calcium signaling pathway | 12 | 0.862 | | |
| Cell cycle | 12 | 0.516 | | |
| Focal adhesion | 28 | 0.466 | 14 | 0.0231 |
| Gap junction | 10 | 0.596 | | |
| GnRH signaling pathway | 11 | 0.651 | | |
| Insulin signaling pathway | 15 | 0.539 | | |
| Leukocyte transendothelial migration | 13 | 0.484 | | |
| MAPK signaling pathway | 30 | 0.187 | 13 | 0.0864 |
| Pathogenic Escherichia coli infection - EHEC | 10 | 0.063 | | |
| Pathogenic Escherichia coli infection - EPEC | 10 | 0.063 | | |
| Phosphatidylinositol signaling system | 10 | 0.525 | | |
| Purine metabolism | 14 | 0.482 | | |
| Pyrimidine metabolism | 11 | 0.151 | | |
| Regulation of actin cytoskeleton | 28 | 0.135 | 11 | 0.134 |
| Tight junction | 11 | 0.897 | | |
| Wnt signaling pathway | 18 | 0.538 | | |
