# Supplementary material for: The Colorectal cancer disease-specific transcriptome may facilitate the discovery of more biologically and clinically relevant information
Source: BMC Cancer. 2010 Dec 20;10:687. doi: 10.1186/1471-2407-10-687 (PMC3018462; doi:10.1186/1471-2407-10-687)
Supplement: Additional file 6 — Pie charts displaying the Colorectal DSA-specific (unique) content (probesets) breakdown for the 5-FU-resistant experiment. A. Based on detected probesets. B. Based on detection + differential expression. [file 1471-2407-10-687-S6.PPTX]

## Slide 1
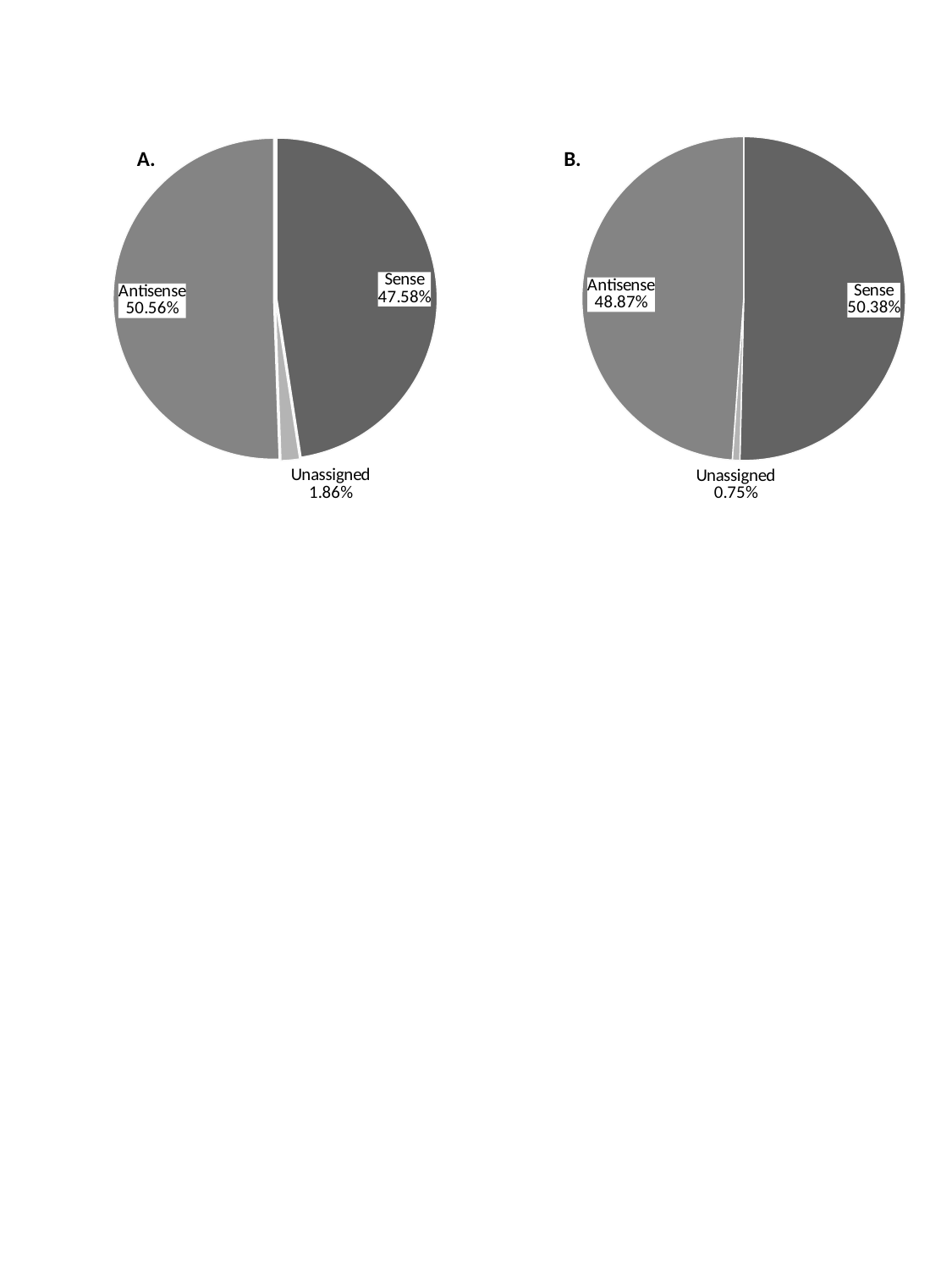

### Chart
| Category |
|---|
### Chart
| Category | |
|---|---|
| Annotated | 894.0 |
| Unannotated | 35.0 |
| Antisense | 950.0 |
### Chart
| Category | |
|---|---|
| Annotated | 67.0 |
| Unannotated | 1.0 |
| Antisense | 65.0 |A.
B.
